# Assessment of Medicalization of Pregnancy and Childbirth in Low-risk Pregnancies: A Cross-sectional Study

**DOI:** 10.30476/IJCBNM.2021.90292.1686

**Published:** 2022-01

**Authors:** Shadi Sabetghadam, Afsaneh Keramat, Shahrbanoo Goli, Mina Malary, Sedighe Rezaie Chamani

**Affiliations:** 1 Student Research Committee, School of Nursing and Midwifery, Shahroud University of Medical Sciences, Shahroud, Iran; 2 Department of Reproductive Health, School of Nursing and Midwifery, Shahroud University of Medical Sciences, Shahroud, Iran; 3 Department of Epidemiology and Biostatistics, School of Public Health, Shahroud University of Medical Sciences, Shahroud, Iran; 4 Department of Midwifery, School of Nursing and Midwifery, Guilan University of Medical Sciences, Rasht, Iran

**Keywords:** Cesarean section, Childbirth, Medicalization, Midwife-led care, Pregnancy

## Abstract

**Background:**

Medicalization may lead to over-testing during pregnancy and increased cesarean section (CS). This study investigated the medicalization of low-risk pregnancies and childbirths in Rasht, Iran.

**Methods:**

In this cross-sectional study, 337 postpartum women completed a demographic questionnaire and the Medicalized Pregnancy and Childbirth checklist. In this study, medicalization indicators were the source of providing prenatal care, prenatal screening for aneuploidy, number of received care, hospitalization before the onset of labor, intrapartum drug use, and CS. Demographic data were reported using descriptive statistics. Chi-square or Fisher’s exact and Man-Whitney tests were used for comparison purposes. Logistic regression was run to determine the medicalization indicators associated with the mode of childbirth.

**Results:**

Of the participants, 82.2% received prenatal care from obstetricians, 85.8% had undergone prenatal screening tests. There was a significant difference between the median number of ultrasound examinations (P=0.006), prenatal screening for aneuploidy (P=0.002), and multivitamin/mineral supplements use (P<0.001), according to the source of providing prenatal care. Of the participants, 67.1% had CS. Women who received prenatal care from obstetricians had about 2.3 times more odds of CS (OR=2.23, P=0.019). Furthermore, with the increased number of ultrasounds, the odds of CS augmented by 25% (OR=1.25, P=0.013). Finally, 26.4% of the participants were hospitalized before the onset of labor; the intervention increased the odds of CS more than twice (OR=2.08, P=0.026).

**Conclusion:**

The study showed a picture of medicalization in low-risk pregnancies. Of the medicalization indicators, the source of providing prenatal care, time of admission, and use of ultrasounds were associated with CS. Midwife-led care could diminish medicalization.

## INTRODUCTION

Medicalization is defined as the use of medical interventions for non-medical problems, which in itself is neither bad nor good. ^1 , 2^ Medical interventions are currently overused in low-risk pregnancies and childbirths, which has led to over-testing during pregnancy, the upward trend in cesarean section (CS), and in turn, increased medical costs. ^3 , 6^

Cesarean section is not the only intervention that medicalizes the pregnancy. Current evidence indicates that pregnancies are increasingly managed, monitored, and often terminated by medical interventions. ^3 , 6^ The World Health Organization (WHO) has recommended that labor induction should not be performed without a clear medical indication as the intervention itself carries a risk of uterine hyper-stimulation, rupture, and fetal distress. ^7^ In addition, the routine use of electronic fetal monitoring, routine active management of care, and use of episiotomies without indication are not advised. Furthermore, increased medicalization of childbirth tends to undermine the laboring woman’s own ability to give birth and negatively impact her labor experience. ^4 , 7^ Reduced interventions improve the quality of care in pregnancy and labor, increases the women’s satisfaction, and makes childbirth a safer experience. ^8 , 9^

Studies showed that unnecessary prenatal interventions such as overuse of ultrasounds, electronic fetal monitoring, induction and augmentation of labor could lead to increased rate of CS; ^10 , 11^ therefore, CS could be the result of a medicalized prenatal and intrapartum care and an important indicator for medicalization of pregnancy, prenatal care, and childbirth. ^3 , 12^ Previous studies are limited to medicalization of childbirth, ^5 , 13^ and few studies have investigated the medicalization of pregnancy and prenatal care in Iran.3 In addition, the rate of CS in Rasht is higher than the national average. ^14^ Therefore, this study aimed to investigate the medicalization of low-risk pregnancies and childbirths in a tertiary referral hospital of Rasht, Iran.

## METHODS

The present cross-sectional-correlational study was conducted between December 2018 and March 2019. The participants were low-risk pregnant women who gave birth at Al-Zahra referral training and research maternity hospital in Rasht, Iran. The women were selected using the convenience sampling method. The inclusion criteria were postpartum women with vaginal delivery or CS and willingness to participate in the study. Pregnant women with a history of infertility, high-risk pregnancy or childbirth (age over 35 years, history of chronic disease like diabetes mellitus, high blood pressure, preeclampsia, and pregnancy or childbirth complications) were excluded from the study. In addition, the participants’ medical records were reviewed to identify high risk mothers and prevent recall bias.

The sample size was determined 334, using a formula by considering a confidence interval of 95%, an acceptable error (d) of 0.05, and the estimated prevalence (P) of 0.33 for the unnecessary CS in Iran. ^15^

$n=\frac{{\left(Z_{1-\alpha /2}\right)}^{2}(\mathrm{pq})}{{\left(d\right)}^{2}}$

Of the 587 deliveries that occurred during the study period, 230 high-risk pregnancies were excluded, and 20 women were not willing to participate. In total, 337 women with low-risk pregnancy took part in the study. The participants completed the research questionnaires on the discharge day. 

Demographic characteristics were collected using a checklist including age, education, husband education, woman’s job, husband’s job, health insurance, complementary insurance, and housing status. Medicalization status was evaluated using the Medicalized Pregnancy and Childbirth checklist that consists of questions about pregnancy and childbirth history and prenatal care information. This checklist was developed and validated by Sedigh *et al*. The validity of this tool was assessed using content and face validity. Total content validity ratio (CVR) and total content validity index (CVI) of the checklist were 0.8 and 0.9, respectively. The reliability was assessed with test-retest. For qualitative variables, they used the Kappa agreement coefficient, and quantitative variables were investigated using a correlation coefficient (P <0.001). The checklist had seven questions to determine the obstetric history of the participants, 11 questions to show the elements of prenatal care, and 15 questions to evaluate intrapartum care. ^3^

Medicalization indicators in this study were receiving prenatal care from an obstetrician, prenatal screening for aneuploidy, number of laboratory tests, number of vitamin-mineral supplements more than recommended in the National guidelines, medication for common pregnancy compliments, number of ultrasounds done, number of non-stress tests, getting iv-line on admission, intrapartum drug use, electronic fetal monitoring, hospitalization before the onset of labor, and CS. We also investigated such interventions as permission to move freely and change position, fundal pressure and episiotomy in the participants who were candidates for vaginal birth.

### Statistical Analysis

Demographic data were reported using descriptive statistics such as frequency, mean, median and standard deviation; Chi-square or Fisher’s exact test was used to evaluate the relationship between obstetrics characteristics, medicalization indicators, and the mode of delivery. Kolmogorov-Smirnoff test was used to examine the normality of the number of received care. Mann-Whitney test were used to compare the number of received care according to the source of providing prenatal care. Also, logistic regression was used to determine the variables associated with the mode of childbirth. All the statistical analyses were performed using SPSS, version 25. A P-value of less than 0.05 was considered statistically significant. 

This study was approved by the Ethics Committee of Shahroud University of Medical Sciences, Iran (ethical approval code: IR.SHMU.REC.1397.062, Date: 2018-07-02). The participants were ensured that their data would remain confidential, and written consent was obtained from them prior to participation in the study. The patients were reassured that unwillingness to take part in the study would not affect their treatment process.

## RESULTS

The mean ages of the participants and their husbands were 29±5.86 and 33.26±6.40 years, respectively. Of the participants, 317 (94.1%) were housewives and 153 (45.5%) had elementary education. Most of the husbands (290; 86.1%) were self-employed and 165 (49%) men had elementary education. Most of the participants (329; 97.6%) had health insurance, but 317 (94%) subjects did not have complementary insurance (Table 1). The majority of the participants had CS 226 (67.1%) and the majority of primiparous women had CS 65 (60.2%). All the participants had prenatal care, which was mostly received from obstetricians (277; 82.2%). Of women, 289 (85.8%) had undergone prenatal screening tests. 282 participants (82.9%) had consumed iron and folic acid supplements and 60 (17.9%) had taken multivitamin-mineral supplements more than recommended in the National guidelines. About one-third of the participants took medications for common problems in pregnancy (114; 33.8%). 

**Table 1:** The participants’ background (N=337)

Variables	Categories	N (%)^a^
Age	18-25	100 (29.7)
	25-30	186 (55.2)
	30-35	51 (15.1)
Women’s education	Elementary	153 (45.5)
	Secondary	133 (39.5)
	University	51 (15)
Woman’s job	Housewife	317 (94.1)
	Employed	20 (5.9)
Husbands’ education	Elementary	165 (49)
	Secondary	132 (39.2)
	University	40 (11.8)
Husbands’ job	Worker/Farmer	25 (7.4)
	Self-employed	290 (86.1)
	Employed	22 (6.5)
Housing Status	Rental	116 (34.4)
	Private	169 (50.2)
	Live with parents	52 (15.4)
Health insurance	Yes	329 (97.6)
	No	8 (2.4)
Complementary health insurance	Yes	20 (6)
	No	317 (94)
Parity	Primigravida	134 (39.8)
	Gravida 2	152 (45.1)
	Gravida 3 and more	51 (15.1)

a : Number and percentages

**Figure 1:**
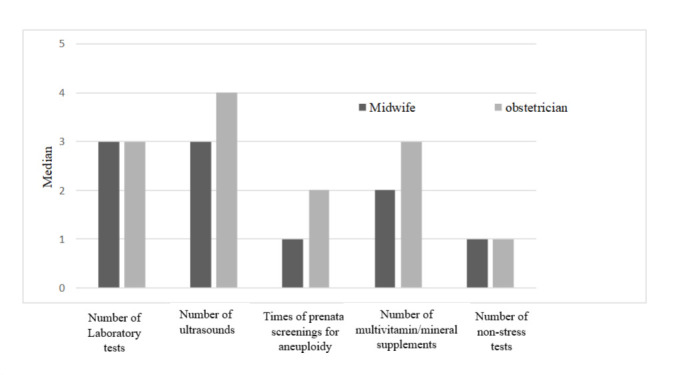
Median number of received care according to source of providing prenatal care

The number of received care had no normal distribution, and Man-Whitney test showed a significant difference between the median of ultrasound examinations (P=0.006), prenatal screening for aneuploidy (P=0.002), and multivitamin-mineral supplements use (P<0.001), according to the source of providing prenatal care. (Figure 1).

89 participants (26.4%) were hospitalized before the onset of labor. Most of them received fetal heart monitoring before admission for labor. For most women, an IV line was established on admission (211; 62.7%). From 186 (55.19%) women who were primarily candidates for vaginal birth, half of them received induction and most of them received pharmacological pain relief. In addition, about half of the participants reported that they had free movement during labor, and half of them were able to change position. For participants who had vaginal birth, episiotomy (97; 87.4%) and fundal pressure during birth (72; 62.9%) were used in the majority of childbirths. Obstetricians and residents were present at birth in 75% of vaginal births.

Evaluation of the relationship between prenatal and intrapartum interventions and the mode of delivery indicated that the source of providing prenatal care was significantly associated with the mode of childbirth in low-risk before prenatal screening for aneuploidy (P=0.002). In other words, the rate of CS was higher in women with low-risk pregnancies who received prenatal care by obstetricians. The time of hospitalization was significantly associated with the mode of childbirth (P=0.001). The rate of CS was higher in women who were hospitalized before the onset of labor signs. Induction with oxytocin performed during labor was significantly associated with the mode of childbirth (P <0.001), and the highest rate of vaginal birth was observed in the group who had received labor induction with oxytocin. Furthermore, there was a significant association between pharmacological pain relief and the mode of childbirth (P<0.001). This relationship revealed that the rate of CS was higher in the group receiving pharmacological pain relief. Artificial rupture of membranes was significantly related to the mode of delivery (P<0.001), and CS rate was higher in the group who had received this intervention. There was a significant association between freedom of movement in labor and the mode of childbirth (P=0.012) (Table 2).

**Table 2:** Relationship between obstetrics characteristics, medicalization indicators, and the mode of delivery

Variables	Categories	Vaginal Birth N=111 N (%) a	CS b N=226 N (%)	P value *
Parity	Primiparous	44 (40.4)	65 (59.6)	0.048
	Multiparous	67 (29.4)	161 (70.6)	X^2^=4.026
Preconception care	Received	7 (18.2)	27 (81.8)	0.078
	Not received	104 (34.3)	199 (65.7)	X^2^=3.521
Source of providing preconception care	Midwife	1 (10)	9 (90)	0.640
	Obstetrician	6 (21.7)	18 (78.3)	X^2^=0.646
Source of providing prenatal care d	Midwife	28 (70)	12 (30)	0.002
	Obstetrician	83 (49.4)	85 (50.6)	X^2^=9.255
Prenatal screening for aneuploidy	Yes	96 (32.9)	194 (67.1)	>0.999
	No	15 (31.9)	32 (68.1)	X^2^=0.017
Medication for common problem in pregnancy	Yes	32 (27.2)	86 (72.8)	0.107
	No	79 (36)	140 (64)	X^2^=2.601
Time of hospitalization	After onset of labor	92 (37.9)	151 (62.1)	0.001
	Before onset of labor	19 (20.2)	75 (79.8)	X^2^=9.783
EFM c on admission	Yes	83 (34.5)	158 (65.5)	0.518
	No	28 (29.2)	68 (70.8)	X^2^=0.587
Induction d	Yes	73 (66.4)	37 (33.6)	>0.001
	No	38 (38.8)	60 (61.2)	X^2^=16.505
Intrapartum pharmacological pain relief d	Yes	14 (29.8)	33 (70.2)	>0.001
	No	97 (60.3)	64 (39.7)	X^2^=32.089
Artificial rupture of membraned	Yes	47 (40.6)	69 (59.4)	>0.001
	No	64 (67.8)	28 (32.2)	X^2^=20.271
Freedom of movement d in labor	Yes	69 (70.4)	29 (29.6)	0.012
	No	42 (38.2)	68 (61.8)	X^2^=6.497
Choice of position in labor d	Yes	82 (66.7)	55 (33.3)	0.063
	No	29 (40.9)	42 (59.1)	X^2^=3.884
**Number of received care**		**Mean±SD**	**Mean±SD**	**P value** **
	Laboratory test	3.23±2.25	3.11±1.22	0.637
	Ultrasound	3.49±1.48	4.12±1.78	0.003
	Vitamin-mineral supplements	2.69±0.85	2.80±0.86	0.319
	Prenatal screenings for aneuploidy	1.45±0.84	1.50±0.81	0.417
	Non-stress tests	3.37±3.98	2.43±3.35	0.94

* Chi-square or Fisher’s Exact test;

** Mann-Whitney test;

a : Number and percentages;

b : Cesarean section;

c : Electronic fetal monitoring;

d : In women who primarily candidate for vaginal birth

Using logistic regression, we assessed the relationship between the mode of delivery and medicalization factors after adjustment for the participants’ age, education, parity, and complementary insurance. Results showed that with women’s advancing age, the odds of CS increased by 6% (OR=1.06, P=0.012). Women who had received prenatal care by obstetricians had about 2.3 times higher odds of CS (OR=2.23, P=0.019). Hospitalization before the onset of labor increased the odds of CS more than twice (OR=2.08, P=0.026). Finally, with increased number of ultrasounds, the odds of CS increased by 25% (OR=1.25, P=0.013; Table 3).

**Table 3:** Logistic regression analysis for the association between medicalization indicators and mode of childbirth

Variables	Univariate Analysis		Multivariate Analysis	
	OR (CI)^a^	P value*	OR (CI)	P value**
Age	1.07 (1.033, 1.12)	<0.001	1.06 (1.01, 1.12)	0.012
Parity	1.62 (1.00, 2.621)	0.046	1.60 (.85, 3.02)	0.145
Education	1.42 (0.906, 2.25)	0.125	1.48 (.86, 2.53)	0.150
Complementary insurance	2.88 (0.82, 10.07)	0.096	2.13 (.53, 8.49)	0.284
Source of providing prenatal care	2.46 (1.372, 4.41)	0.003	2.23 (1.13, 4.38)	0.019
Time of hospitalization	2.48 (1.38, 4.43)	0.002	2.08 (1.09, 3.97)	0.026
Number of laboratory tests	0.95 (0.83, 1.09)	0.539	.90 (.76, 1.06)	0.206
Number of ultrasounds	1.26 (1.08, 1.47)	0.002	1.25 (1.04, 1.49)	0.013
EMF^b^ on admission	0.81 (0.48, 1.37)	0.444	.92 (.50, 1.68)	0.789

* Univariate logistic regression analysis;

** multivariate logistic regression analysis;

a : Odds ratio (Confidence interval);

b : Electronic Fetal monitoring

## DISCUSSION

This study provided a picture of the medicalization in low-risk pregnancies and childbirths in Rasht, Iran. We also found that some medical interventions like receiving care from an obstetrician, hospitalization before the onset of labor, and increased number of ultrasounds could lead to CS.

Regarding medicalization of prenatal care, although all the participants had a low-risk pregnancy, the majority of them received prenatal care from an obstetrician. In addition, the current study revealed that receiving care from an obstetrician was associated with CS. Midwives often use the social model of care focusing on physiology, and obstetricians use the medical model of care focusing on medical interventions. ^16^ Therefore, one of the indicators for medicalization of pregnancy is receiving care from an obstetrician instead of a midwife for low-risk pregnancies. Previous findings also support a strong link between midwifery-led care for pregnant women and reduced labor and birth interventions, which has important benefits and causes no adverse outcomes for mothers and newborns. ^17 - 19^ Although midwives are the first care providers for low-risk pregnant women in most countries, and since there is no functional referral system in the health sector of Iran, ^20^ many low risk pregnancies and childbirths are not managed by midwives. ^21^ Therefore, childbirth, which is a physiological event, has turned into a medical procedure. 

In addition to the results showing lower rates of CS and fewer ultrasound examinations in participants who received prenatal care from midwives, they also took fewer multivitamin/mineral supplements and underwent fewer prenatal screenings than those who received care from obstetricians. Our results showed that about 18% of the participants had used multivitamin-mineral supplements more than recommended in the National guidelines. Although adequate intake of iodine, folic acid, calcium, and iron must be ensured in pregnancy, ^22^ multivitamin and mineral supplements in pregnancy impose unnecessary costs ^23^ and overconsumption of these supplements may not be safe for low-risk pregnancies. ^24^ Results revealed that most of the participants used prenatal screening tests. Similarly, prenatal screening tests have become widely common among pregnant women in other countries. ^25 , 26^ Prenatal screening for trisomies and neural tube defects was primarily recommended only for high-risk pregnancies, but in many countries, including Iran before 2020, it is recommended for low-risk pregnancies as well. Women should be informed of the benefits and limitations of prenatal screening tests to enable them to make informed decisions. Doing this intervention for the general population raises many ethical issues. ^27^ Overuse of prenatal screening tests in the low-risk population of this study may show the growing obstetric-led care model in our society. The results showed that the number of ultrasounds is associated with CS. It might be explained by the fact that most participants receive care from obstetricians, and obstetricians routinely do ultrasounds for pregnant women at their offices. Huang et al. (2012) reported a statistically significant association between antenatal ultrasound scans and CS. ^10^ The safety of ultrasound in pregnancy is unclear, ^28^ and there is still controversy about whether the routine use of ultrasound during pregnancy brings benefits to the mother and baby in low-risk pregnancies. ^29^

Regarding medicalization of intrapartum care, about one-third of the participants were hospitalized before the onset of labor, and the results showed that women who were hospitalized before the onset of labor signs were at a greater risk for CS. This finding is consistent with that of Colaka and Can (2020) who found that the CS rate was higher in the women admitted to the hospital in the latent phase. In addition, obstetricians prefer to conduct CS before the onset of labor. ^30^ Elective CS has a high risk of developing neonatal respiratory morbidities when compared to vaginal birth. ^31^ The highest rate of vaginal birth was observed in the group who had received labor induction with oxytocin. This finding is contrary to those of Sedigh *et al*. (2017). ^3^ A possible explanation for our result might be the routine use of oxytocin for vaginal birth candidates in our setting.

The results showed that participants who received pharmacological pain relief in labor had more CS than those who did not. The drug that is commonly used in our setting is pethidine. Nunes and Primo (2019) reported that pethidine was safe when used during labor,32 but some studies showed that it might have maternal and neonatal complications. ^33 , 34^ This inconsistency may be due to the sample size of the studies or the dose of pethidine. In addition, participants who had artificial rupture of membrane (amniotomy) had more CS than those who did not. Studies had a controversy about the effect of amniotomy on the duration of labor and CS rate. Although in the present study we did not include the time of artificial rupture of membrane, early amniotomy may lead to the increased rate of CS. ^35 - 37^ In our study, the highest rate of vaginal birth was observed in the group who had freedom of movement in labor. Prosser *et al*. (2018) found that freedom of movement was a promoting factor for normal birth. ^38^

Furthermore, in the present study, although women with high-risk pregnancies were excluded, findings showed that most of them received intrapartum care in the presence of the resident or obstetrician. A recent study found that receiving intrapartum services from midwives could reduce the CS rate compared with obstetrician-led care in low-risk pregnancies and childbirths. ^19^ In addition, more than half of primiparous participants had CS. Cesarean section is a lifesaving intervention which reduces perinatal mortality and morbidity, but studies have demonstrated that CS rates above 15% are not associated with lower maternal and neonatal mortality and morbidity. ^39 , 40^

Strengths and Limitations

This study investigated the medicalized prenatal care and childbirth in low-risk pregnancies. The results of the current study could be useful in the planning of programs to improve care of low-risk pregnancies and childbirths. The findings may be generalized to similar low-risk pregnant populations. This study has some important limitations as well. First, the cross-sectional nature of the study did not allow for the establishment of causal relationships among the study variables. Second, this study was done in a single public hospital; hence, further studies using a more representative data for public and private hospitals in Iran is required. Third, although we did our best to exclude the complicated deliveries, our information about the childbirth process was from medical records, not the researcher’s observation, so our information about the childbirth process may not be completely reliable. Fourth, 129 participants had a previous CS, and these women are routinely candidates for a repeated CS in our setting and vaginal birth after CS is not a common choice for them; therefore, the issue could affect the conclusion that CS will be higher when the source of providing prenatal care is an obstetrician.

## CONCLUSION

Our results showed a picture of medicalization in the care of low-risk pregnancies and childbirths in a referral maternity hospital. Of the medicalization indicators we assessed in this study, the source of providing prenatal care, time of admission, and use of ultrasounds were associated with CS. Receiving prenatal care from an obstetrician could increase the odds of CS, while midwife-led care could decrease medicalization of pregnancy and childbirth. The known risks and benefits of prenatal care interventions and mode of childbirth should be described for pregnant women to enable them to make informed decisions with the help of health care providers. Community-based studies are needed to investigate the factors related to medicalization of pregnancy and childbirth.

## ACKNOWLEDGEMENT

This study was supported by grant No 9761 from Shahroud University of Medical Sciences. We thank the Research Deputy of Shahroud University of Medical Sciences for providing facilities and financial supports. We also thank the staff of Guilan University of Medical Sciences who kindly collaborated in this study.

**Conflict of Interest:** None declared
